# A Microfinance Intervention With or Without Peer Support to Improve Mental Health Among Transgender and Nonbinary Adults (the Creating Access to Resources and Economic Support Study): Protocol for a Randomized Controlled Trial

**DOI:** 10.2196/63656

**Published:** 2024-08-26

**Authors:** Tonia C Poteat, Sari L Reisner, Andrea L Wirtz, Larissa Jennings Mayo-Wilson, Carter Brown, Wiley Kornbluh, Ash Humphrey, Nancy Perrin

**Affiliations:** 1 Duke University School of Nursing Durham, NC United States; 2 Department of Epidemiology University of Michigan School of Public Health Ann Arbor, MI United States; 3 The Fenway Institute Boston, MA United States; 4 Department of Epidemiology Johns Hopkins Bloomberg School of Public Health Baltimore, MD United States; 5 Department of Health Behavior Gillings School of Global Public Health University of North Carolina Chapel Hill, NC United States; 6 National Black Trans Advocacy Coalition Carrollton, TX United States; 7 Johns Hopkins School of Nursing Baltimore, MD United States

**Keywords:** clinical trial, depression, anxiety, peer support, minority stress, cash transfer, COVID-19

## Abstract

**Background:**

Transgender and nonbinary (TNB) people experience economic and psychosocial inequities that make them more likely to be subject to financial and mental health harms exacerbated by the COVID-19 pandemic. Sustainable, multilevel interventions are needed to address these harms. The onset of the COVID-19 pandemic galvanized many TNB-led organizations to provide emergency financial and peer support for TNB people negatively impacted by the pandemic. However, the efficacy of these interventions has not been evaluated. The Creating Access to Resources and Economic Support (CARES) study seeks to assess the efficacy of feasible, acceptable, and community-derived interventions to reduce economic and psychological harms experienced by transgender people in the wake of the COVID-19 pandemic.

**Objective:**

The study aims to (1) compare the efficacy of microgrants with peer mentoring with that of microgrants without peer mentoring in reducing psychological distress, (2) examine mechanisms by which microgrants with or without peer mentoring may impact psychological distress, and (3) explore participants’ intervention experiences and perceived efficacy.

**Methods:**

We will enroll 360 TNB adults into an embedded, mixed methods, 3-arm, and 12-month randomized controlled trial. Participants will be randomized 1:1:1 to arm A (enhanced usual care), which will receive a single microgrant plus monthly financial literacy education, arm B (extended microgrants), which will receive enhanced usual care plus monthly microgrants, or arm C (peer mentoring), which will receive extended microgrants combined with peer mentoring. All intervention arms last for 6 months, and participants complete semiannual, web-based surveys at 0, 6, and 12 months as well as brief process measures at 3 and 6 months. A subset of 36 participants, 12 (33%) per arm, will complete longitudinal in-depth interviews at 3 and 9 months.

**Results:**

Full recruitment began on January 8, 2024, and, as of July 26, 2024, a total of 138 participants have enrolled. Recruitment is expected to be completed no later than March 31, 2025, and the final study visit will take place in March 2026.

**Conclusions:**

This national, web-based study will demonstrate whether an intervention tailored to reduce material hardship and improve peer support among TNB adults will reduce psychological distress. Its equitable, community-academic partnership will ensure the rapid dissemination of study findings.

**Trial Registration:**

ClinicalTrials.gov NCT05971160; https://clinicaltrials.gov/study/NCT05971160

**International Registered Report Identifier (IRRID):**

DERR1-10.2196/63656

## Introduction

### Financial and Mental Health Inequities

The COVID-19 pandemic has had devastating impacts on transgender and nonbinary (TNB) people. TNB people are a National Institutes of Health–designated health disparity population [1] who experience economic and psychosocial disadvantages that make them particularly vulnerable to pandemic-related harms [2]. Baseline data from an observational cohort of >1500 transgender women indicated elevated prepandemic levels of poverty (46% vs 11%), food insecurity (48% vs 11%), and survival sex work (21% vs 1%) compared with the general US population [2]. Participants also reported high levels of psychological distress (27%), traumatic stress symptoms (41%), and suicidal ideation (28%), all of which can be exacerbated by pandemic-related stress [2]. Another prepandemic study of 850 transgender men also identified high levels of psychological distress (20%), hazardous alcohol use (59%), and polysubstance use (20%) [3]. These prepandemic vulnerabilities are consistent with a nationally representative sample of TNB adults in which 48% were living in poverty and 42% had a history of prior suicide attempts [4].

A national poll of 7000 adults conducted after the onset of the COVID-19 pandemic found that twice as many TNB people became unemployed and 5 times as many TNB people received a pay cut due to the pandemic compared with the general population [5]. A later poll found that TNB people were 125% more likely to have reduced work hours since states reopened after the COVID-19 lockdowns expired [6]. A longitudinal study of 208 TNB people found significant increases in psychological distress, depression, and anxiety after the pandemic onset compared with before the pandemic [7]. A mixed methods study of Latina transgender women found a significant decline in mental health, with the psychological health in the “likely to be well” range falling from 93% (prepandemic) to 50% (after pandemic onset) [8]. Qualitative findings indicated that much of this psychological distress was related to pandemic-induced material hardship [8]. In summary, TNB people experienced financial and psychological inequities before the pandemic and were highly vulnerable to long-term negative economic and mental health consequences of the COVID-19 pandemic.

Pandemic-induced material hardship has been associated with poor mental health globally [9]. At the national level, economic recession has been associated with psychological distress [10]. At the neighborhood level, poverty has been associated with depression and anxiety [11]. At the individual level, material hardship (eg, difficulty paying utility bills, rent, or mortgage or fear of running out of food) has been associated with poor mental health [12,13] and the worsening of existing mental health conditions [14] independent of other measures of socioeconomic status. Material hardship increases exposure to stressors (eg, inability to pay for basic needs) and stressful events (eg, eviction) while also hindering the ability to cope with stress [15,16]. A recent cross-sectional study of 849 transgender people using structural equation modeling found that socioeconomic loss partially mediated the relationship between pandemic-related societal changes (eg, social isolation) and poor mental health [17].

### Microfinance Interventions

Microgrants (also called cash transfers) provide a small monetary amount to individuals or families, which they do not have to repay. They can be considered structural interventions because they alter the economic conditions within which health outcomes are produced or reproduced [18]. Cash transfers have been extensively studied in large-scale cluster randomized trials in low- and middle-income countries [19-21] where positive effects on mental health were found [21-24]. A randomized trial with unhoused individuals in Vancouver found that cash transfer recipients accessed stable housing more quickly than controls and increased spending on food, clothing, and rent while reducing spending on alcohol and drugs [25].

Of the 2 major cash transfer programs in the United States, namely the Alaskan National Petroleum Reserve Impact Grant Program [26] and the North Carolina Eastern Band of Cherokee casino grants [27], only the Cherokee program has been evaluated. Several studies have demonstrated positive mental health effects of the Cherokee program [27,28]. While these data provide an important premise for the psychological benefits of cash transfers in the United States, these programs provide ongoing, long-term, and annual revenue generated from business enterprises and do not address whether short-term, targeted microgrants, provided incrementally over several months, can mitigate the long-term negative mental health effects of a pandemic crisis such as the COVID-19 pandemic. Data are needed not only to understand whether short-term microgrants are effective in improving TNB mental health inequities exacerbated by the pandemic but also to assess the mechanisms that may account for their effects.

### Peer Support Interventions

Social distancing and isolation, the key aspects of the COVID-19 pandemic response, have been linked to poor mental health [29]. A longitudinal study of 208 TNB adults found that the loss of transgender-specific support during the pandemic was associated with greater intrapandemic psychological distress [7]. Social support and community connection are known to facilitate mental health recovery after crises [30]. Studies conducted before the pandemic indicate that social support via transgender community connectedness can reduce suicidal ideation, anxiety, and depression and buffer the mental health effects of stressors among transgender people [31-34]. These data suggest that supportive transgender community connection (ie, peer support) may reduce the psychological distress associated with the pandemic.

Peer mentoring is a form of peer support in which a trained peer promotes skill building in a mentee [35]. Evidence suggests that peer support alone is unlikely to improve mental health in the context of economic challenges [36]. Combining peer mentoring with microgrants may be more beneficial for mental health than providing microgrants alone. However, studies of combined financial and peer support interventions are limited, and none have examined psychological outcomes [37-39]. Given the lasting multilevel harms caused by the COVID-19 pandemic and the associated economic downturn, combining an intervention that directly addresses material hardship (structural) with one that provides peer mentoring support (interpersonal) may be the most effective.

### Community Leadership and Engagement

The COVID-19 pandemic galvanized transgender-led organizations, such as the National Black Trans Advocacy Coalition (BTAC), to provide support for impacted TNB people [40]. BTAC provides peer mentoring as well as referrals and support for health, housing, and employment for TNB people. During the early phase of the COVID-19 pandemic, BTAC provided TNB applicants with a onetime US $125 microgrant. They awarded >1200 microgrants and reached >5000 TNB people with health, housing, or employment referrals and peer support [40]. However, the efficacy of these community-led social and structural interventions has not been evaluated. Strong community-academic partnerships with organizations such as BTAC can ensure that community-derived, relevant, feasible, acceptable, and effective interventions are identified and translated into sustainable change.

### Theoretical Framework

**Figure 1:**
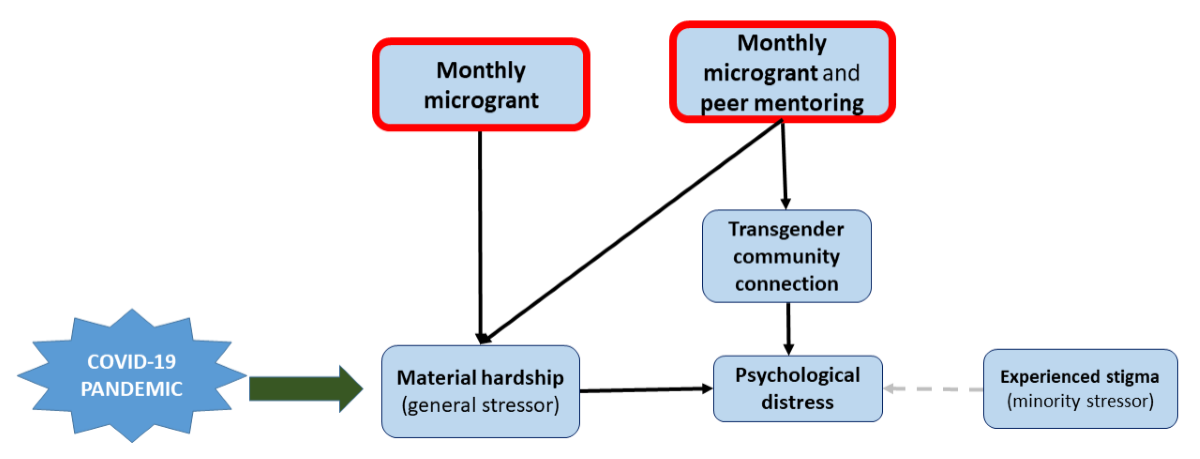
A theoretical model for the effects of the COVID-19 pandemic on material hardship and psychological distress among transgender and nonbinary people who experience minority stress.

The Gender Minority Stress and Resilience (GMSR) model [41] describes the way interventions are expected to act via multiple levels of the National Institute on Minority Health and Health Disparities Research Framework [42] to mitigate lasting pandemic-induced harms. Briefly, the GMSR model posits that TNB people experience a combination of general (eg, material hardship) and minority (eg, stigma) stressors. The excess minority stressors combined with general stressors, experienced regardless of gender identity, lead to mental health inequities. The model posits that transgender community connectedness will mitigate minority stress and improve mental health. The Creating Access to Resources and Economic Support (CARES) study (Figure 1) will test whether addressing a general structural-level stressor (material hardship) alone or in combination with social support (ie, peer mentoring to increase transgender community connection) at the interpersonal level will improve mental health and COVID-19 risk reduction behaviors without intervening directly on minority stressors [43].

The CARES study seeks to assess the efficacy of microgrants with or without peer support to reduce economic and psychological harms experienced by TNB people in the wake of the COVID-19 pandemic. The CARES study builds on TNB community–led interventions, leverages the expertise of an existing TNB community advisory board (CAB), and extends ongoing community-academic partnerships with BTAC. This paper outlines the protocol for the CARES study.

### Study Aims and Hypotheses

The CARES study has 3 aims and 3 hypotheses (Textbox 1).

**The Creating Access to Resources and Economic Support (CARES) study aims and hypotheses.**

**Aims and hypotheses**

- Aim 1 is to compare the efficacy of monthly microgrants, with or without peer mentoring, to reduce psychological distress among transgender and gender nonbinary (TNB) adults, relative to the receipt of a single microgrant. This includes hypothesis 1, that the receipt of monthly microgrants, with or without peer mentoring, will significantly reduce psychological distress scores compared with the receipt of a single microgrant, and exploratory hypothesis 1.1, that the receipt of monthly microgrants with peer mentoring will significantly reduce psychological distress scores compared with the receipt of monthly microgrants without peer mentoring.
- Aim 2 is to examine mechanisms by which monthly microgrants, with or without peer mentoring, impact psychological distress among TNB adults. This includes hypothesis 2, that material hardship will mediate relationships between the receipt of monthly microgrants and psychological distress scores, and hypothesis 3, that transgender community connectedness will mediate relationships between the receipt of monthly microgrants with peer mentoring and psychological distress scores.
- Aim 3 is to qualitatively explore intervention experiences and perceived efficacy with TNB adults.

## Methods

### Trial Design

**Figure 2:**
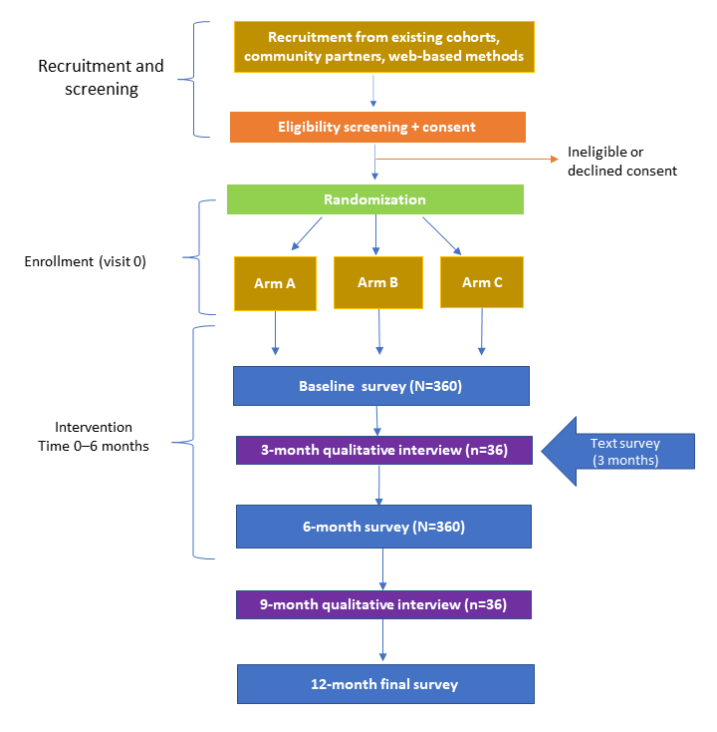
Diagram of study flow for the Creating Access to Resources and Economic Support (CARES) study.

This study uses an embedded mixed method design for a 3-armed, randomized controlled trial with 360 transgender adults. Participants are randomized to 1 of 3 arms: arm A (enhanced usual care), which includes monthly financial education videos for 6 months and a single microgrant of US $150 at baseline; arm B (the extended microgrant arm), which includes enhanced usual care components with the addition of monthly microgrants of US $150 for a total of US $900 over the course of 6 months; or arm C (the peer mentoring arm), which includes the extended microgrant arm components with the addition of a structured peer mentoring intervention for a total of 6 months. All study procedures are self-administered over the web or interviewer administered over the telephone, as described in the Study Procedures section (Figure 2).

Potential participants complete an interest form that is reviewed by the participant engagement coordinator (PEC) to exclude bots. Once the interest form is reviewed and approved by the PEC, the participant is sent an individualized link to a web-based prescreening form. Participants deemed eligible based on the prescreening form are then scheduled for final eligibility screening by telephone. Eligible individuals take part in informed consent procedures. Individuals who provide informed consent are then stratified by gender identity to ensure balance across gender groups (transgender man, transgender woman, and gender nonbinary) and then randomized to 1 of the 3 study arms (Table 1). Once the randomized participant completes the baseline survey, they are considered fully enrolled.

Thirty-minute survey questionnaires are conducted at 0, 6, and 12 months. The 6-month survey will assess changes in outcome measures from baseline to the end of the intervention. The 12-month survey will assess for changes in outcomes since the intervention ended. Participants will also complete brief (3-5 minutes) process measures at 3 and 6 months that assess how microgrant funds were spent, participation in peer mentoring sessions, and engagement with financial education videos. During the course of the intervention, a research assistant makes monthly outreach calls to confirm identity and contact information for sending microgrants and study incentives. A subset of 36 participants will be invited to complete in-depth interviews (IDIs) in the middle of the intervention (3 months) and after the intervention (9 months) to explore changes in experiences and perceptions during and after the assigned intervention. Participants will be purposively selected for interviews using maximum variation sampling by gender identity, racial identity, and geography.

**Table 1:** Overview of the study arms and planned sample sizes (N=360).

Intervention for each study arm^a^	Peer mentoring	Monthly microgrant	Sample size, n (%)
Arm A: enhanced usual care (a single US $150 microgrant)	No	No	120 (33.3)
Arm B: extended microgrant (monthly US $150 microgrant × 6 months)	No	Yes	120 (33.3)
Arm C: peer mentoring (monthly US $150 microgrants × 6 months + peer mentoring)	Yes	Yes	120 (33.3)

^a^All study participants will receive brief financial education videos each month for 6 months.

### Ethical Considerations

This study has received ethics approval from the Duke University Medical Center Institutional Review Board (IRB; Pro00113319). The Duke IRB serves as the IRB of record for all partner institutions in this collaborative study. Before enrollment, all participants complete a detailed informed consent process, including an explanation of their ability to opt out of participation and a consent quiz to ensure the understanding of study procedures. All participants electronically sign a written informed consent document. This protocol was registered in ClinicalTrials.gov (NCT05971160) on July 24, 2023.

Data are collected using REDCap (Research Electronic Data Capture; Vanderbilt University), a secure, web-based platform designed for health research. The website maintains stringent levels of encryption specifically designed to meet and exceed research standards for Internet security as well as IRB standards for the protection of research participants and electronic records. Servers are protected by high-end firewall systems and uses transport layer security (TLS) encryption for all transmitted data. Participant identifiers are stored separately from survey data; and only deidentified datasets are downloaded onto the secure cloud service, OneDrive, for analyses.

Participants are compensated US $50 for completion of the baseline and 6-month survey, respectively. They receive $10 for completion of the 3-month process measures; and $70 for competing the final 12-month survey. Participants who complete IDIs are compensated $50 for each interview.

### Eligibility Criteria

Study participants must meet the following self-reported eligibility criteria: (1) age ≥18 years,; (2) gender identity different from the sex assigned on the original birth certificate (inclusive of TNB people); (3) ability to provide informed consent in English; (4) access to a mobile phone and email; (5) a score >0 on the material hardship index, which indicates exposure to at least 1 material hardship; and (6) willingness and ability to provide some form of photo ID at enrollment.

### Sample Sizes

#### Quantitative Sample

Statistical power was estimated using the mean and SD from the Leading Innovation in Trans Equity (LITE) study, a longitudinal study with 1273 transfeminine individuals [44]. In this sample, the mean score on the 6-item Kessler Psychological Distress Scale (K6) was 10.67 (SD 5.68). When stratified by food insecurity (a type of material hardship), the difference in mean K6 scores between participants experiencing food insecurity and participants not experiencing food insecurity was 20%. Therefore, we considered a ≥20% improvement in either the extended microgrant or the peer mentoring arm compared to the enhanced usual care arm at 6 months as a clinically meaningful effect size.

Assuming a baseline mean for the K6 of 12.15 (SD 5.83) for all 3 groups, no change in the enhanced usual care arm, a 20% improvement over time in either intervention group (mean 9.72 at 6 months), and an α of .05, power to detect a significant group-by-time interaction is 0.89 with 110 (33.3%) individuals per group (n=330), 0.92 with 120 (33.3%) individuals per group (n=360), and 0.94 with 130 (33.3%) individuals per group (n=390). On the basis of the power analysis, we selected a final sample size of 360, with 120 (33.3%) individuals per group. With this sample size, we will retain sufficient power (0.82) even if we observe a 17% difference. We plan to recruit 400 individuals to allow for a 10% attrition rate over the course of the study. However, a sample size as small as 320 (20% attrition) will still provide statistical power that exceeds 80%.

#### Qualitative Sample

Sample sizes for qualitative research aim to include enough participants to reach the point at which no new, relevant information is gleaned from continued data collection, a concept known as saturation [45]. Studies indicate that saturation often occurs within the first 12 interviews, although early themes can be identified with as few as 6 interviews [46,47]. To facilitate reaching saturation within each arm, we will conduct IDIs with 12 participants per arm (n=36).

### Study Procedures

#### Recruitment

A growing body of research indicates that digital research studies that recruit through web-based and social media advertisements are highly susceptible to fraudulent participation by bots or enrollment by ineligible participants seeking study compensation [48,49]. Financial compensation for study participation has been found to increase the risk of participant deception about study eligibility [50]. However, failure to compensate participants for the burden of research engagement can be considered unethical [51,52]. Given that our study intervention includes microgrant payments, it would be impossible to enroll participants without providing them with money. Therefore, to reduce the potential for fraudulent enrollment, the study team has implemented a tailored recruitment strategy as well as several evidence-based screening strategies [49,53-57] to ensure enrollment only by eligible participants.

**Figure 3:**
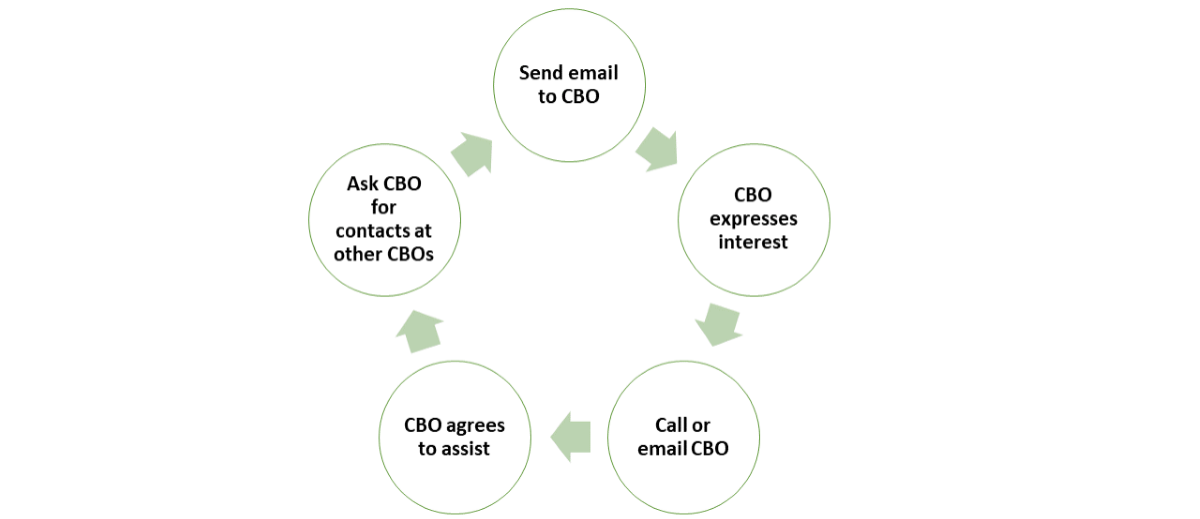
Strategy for community-engaged study recruitment with community-based organizations (CBOs).

The study team leverages community partnerships and existing web-based observational cohorts of TNB adults for recruitment. BTAC, which is based in Texas and has reached >5000 TNB people with services, has taken a leadership role in recruiting potential participants through their extensive community networks. In addition, the study team has developed a database of numerous transgender-led and transgender-focused community organizations across the country, whom we are contacting to share information about the study (Figure 3). If we are unable to reach recruitment goals through these community-engaged strategies, participants in recent and ongoing TNB-focused studies led by our study team, for example, the LITE study (N>1200) and the LEGACY study (N=2011), who have consented to be contacted for future research will be invited to participate in this study [44,58].

#### Screening and Enrollment

Interested potential participants are directed to the CARES study web page for further information and to complete an interest form. The PEC reviews the email address from the interest forms as well as the encrypted IP address for any indication of fraud and then sends a personalized link to the prescreener, which asks questions to assess eligibility for the study, as well as collects a phone number, an email address, and best times for contact. Participants who are prescreened as eligible receive an email informing them that study staff will call or text them to schedule a telephone appointment for enrollment. They are also offered the opportunity to book a specific time using a calendar link. At the scheduled appointment time, the PEC calls to complete the final eligibility screening and conducts the informed consent process. Eligible participants who consent to enroll are randomized to one of the study arms using stratified randomization by gender identity (transgender men, transgender women, and gender nonbinary) to facilitate assessment for gender differences. Once randomized, participants are sent a link to the baseline survey. Participants are considered fully enrolled once the baseline survey is complete.

#### Retention

Upon enrollment, participants are asked to provide extensive contact information, including phone numbers, email addresses, and contact person and number in case study staff are unable to reach them. A dedicated staff member will call participants each month to confirm or update their contact information and will also confirm their identity using date of birth to ensure microgrant payments are being sent to the enrolled individual. The study database has been programmed to automatically send an email and text to each participant when they are due for data collection. Study staff follow up with telephone calls to participants who do not complete the survey within 10 to 15 days after the automated message is sent. We have hired study staff from the TNB community to foster cultural competence and optimize acceptability to participants. We anticipate a 90% retention rate over the 12-month study based on our experience retaining 90% of participants over 12 months in the LITE study.

#### Quantitative Data Collection

All participants complete a 30-minute self-administered web-based survey at baseline and every 6 months for a total of 3 full surveys over the course of 12 months. To reduce bias associated with literacy and technology challenges, interviewer-administered surveys will be offered to all participants at enrollment. We have successfully used this approach for our COVID-19 testing study of >2000 TNB adults across the United States. Study participants will also complete a brief (≤10 minutes) SMS text message survey with only process measures (eg, number of financial education videos viewed, number of peer mentoring sessions attended, and how microgrant funds were spent) at 3 months. These process measures will also be incorporated into the 6-month survey.

A summary of key study measures and their reported internal consistency assessed by Cronbach α, where available, are presented in Table 2. To facilitate data sharing and population comparisons, primary outcome and potential mediator measures are drawn from the PhenX Toolkit Social Determinants of Health [59] and Mental Health Collections and the National Institutes of Health Public Health Emergency and Disaster Research Response [60]. The primary mental health outcome measure is the K6*,* a 6-item Likert scale of psychological distress [61]. K6 is a validated, brief, and self-report instrument that is low burden for investigators and participants [62]. It can easily be self-administered via the internet and has performed well in prior studies with TNB people [63-65]. The summary scale score ranges from 0 to 24, with scores ≥5 consistent with moderate distress [66] and scores ≥13 consistent with significant distress [61]. Higher scores on the K6 have been associated with an increased risk of all-cause mortality [67]. Reductions in K6 scores have correlated with reductions in symptoms of anxiety and depression [68].

**Table 2:** Key study measures.

Construct		Instrument	Variable type	Source	Cronbach α
**Mental and behavioral health**					
	Psychological distress	Kessler 6	Primary outcome	Kessler et al [69], 2002	0.83 [68]
	Substance use disorder	10-item Drug Abuse Screening Test	Exploratory outcome	Yudko et al [70], 2007	0.94
**Economic hardship**					
	Material hardship	Material hardship index	Potential mediator	Oulette et al [71], 2004	Not applicable
**Social support**					
	Peer social support	Transgender community connectedness	Potential mediator	Testa et al [41], 2015	0.90
**Discrimination**					
	Gender-related discrimination	Gender Minority Stress and Resilience	Potential confounder	Testa et al [41], 2015	0.90
	Intersectional discrimination	Intersectional Discrimination Index–Major	Potential confounder	Scheim and Bauer [72], 2019	0.72

The primary economic hardship measure is a 7-item material hardship index used in the Coronavirus Health and Impact Survey V0.2 [73]. This index from the Disaster Research Response has been recommended by the US Department of Health and Human Services and included in the federal Survey of Income and Program Participation by the US Census Bureau [71,74]. The primary social support measure is the validated transgender community connectedness scale, a 5-item subscale of the GMSR measure [41].

While extant data indicate microgrants do not increase substance use [25], we have included the 10-item Drug Abuse Screening Test as a validated screening tool to assess any changes in substance use over the course of the study [75]. None of the CARES study interventions are hypothesized to affect experiences of discrimination. However, experiences of discrimination (eg, employment discrimination) may be associated with economic hardship as well as with mental health; therefore, we have included discrimination measures as potential confounders. Gender-related discrimination is a 5-item subscale of the GMSR measure [41] included to assess experiences related to TNB identity; and the Intersectional Discrimination Index-Major is a 13-item subscale of the Intersectional Discrimination Index [72] included to assess experiences related to any aspect of the participants’ identity.

#### Qualitative Data Collection

Qualitative research is the most appropriate for eliciting detailed accounts of participant experiences and the perceived efficacy of the interventions. The open-ended nature of IDIs provides the opportunity to more deeply explore issues relevant to the study aims [76]. Prior studies have demonstrated how embedding qualitative data collection within a randomized trial enriches the understanding of how and why the interventions work or do not work [77,78]. Longitudinal interviews are an important means by which to study how participants experience, interpret, and respond to their assigned intervention over time.

We will conduct one-on-one IDIs at months 3 and 9 to capture intraintervention and postintervention experiences and perspectives. Interviews will take place using a Health Insurance Portability and Accountability Act–compliant videoconference platform. Our team has successfully collected qualitative data remotely using this method [79]. We will use stratified sampling [80] to select participants who vary by gender identity within each study arm. We will seek variability by race, age, and geography. Each IDI will last approximately 1 hour with an interviewer who has been trained in qualitative research methods and has experience conducting research with TNB people.

A topical guide will structure the interview. Open-ended questions, followed by prompts, as needed, will be used to elicit participant narratives. The initial IDIs will begin by exploring the participants’ lives, stressors, and coping strategies. The interviewer will then guide the discussion toward participants’ experiences of the intervention (eg, how they spend the microgrants and how they relate with their peer mentor) and how they perceive their financial situation and mental health to have changed or not have changed since the intervention began (ie, interim perceived efficacy). Follow-up IDIs will explore how their financial situation and mental health have changed since the intervention ended and discuss their retrospective reflections on the intervention. This qualitative longitudinal design will also enable the study team to clarify any unclear or incomplete responses in the prior IDI in follow-up interviews. All interviews will be digitally audio recorded and transcribed verbatim by a professional transcription company. Interviewers will write field notes and narrative summaries after each interview that will supplement the transcripts. During biweekly meetings, the study team will review field notes, transcripts, and summaries; discuss emerging themes; and revise interviewing and coding strategies, as appropriate.

### Interventions

#### Comparison Intervention: Enhanced Usual Care for All Study Arms

Enhanced usual care interventions include a single microgrant of US $150 provided via virtual or physical Mastercard (Mastercard Inc). In addition, each participant will receive a link to 1 financial education video lasting 5 to 10 minutes each month for 6 months. Prior research identified financial education as an important need for TNB people [81,82]. Providing financial education to every participant in all arms will allow the study team to distinguish the effects of financial education (offered to everyone) from the effects of peer mentoring (offered in only 1 arm). Each video (Table 3) is based on content from a financial literac*y* intervention developed specifically for TNB adults by members of our research team (NCT04275310).

**Table 3:** Financial education video topics and knowledge objectives by the month of intervention.

Topic	Title	Knowledge objective
1	Protecting Yourself & Your Money	Identify and avoid predatory loans, financial abuse, and coercion
2	Income Generation	Identify job opportunities and considerations for the gig economy
3	Banking	Select and open a bank account
4	Budgeting	Develop a personal budget to meet goals
5	Credit and Loans	Build and maintain good credit
6	Transgender Financial Advisor	Navigate financial systems as a transgender person

#### Experimental Intervention: Extended Microgrants for Arms B and C

Participants in arms B and C will receive US $150 per month for 6 months, totaling US $900, via a virtual or physical Mastercard, as outlined for enhanced usual care. During each month of the intervention, participants will receive a phone call from the study team and will be required to respond to verify their date of birth and contact information. After verification, funds will be added to the participants’ accounts. Date of birth verification will be the only condition required for subsequent microgrant funds. The study team disburses funds monthly for 6 months in the intervention arms to be consistent with the frequency and duration of interventions in prior research and to align with what would be the most sustainable after the study.

In the absence of prior US data on microgrants, the study team explored existing data to determine the amount for each microgrant. The median annual income of LITE participants was less than the federal poverty level at the time (US $12,760 for an individual). Therefore, a microgrant of US $150 represents 15% of their monthly income. This is approximately the proportion of income that the average US household spends on food. Because food insecurity is an important element of material hardship, a US $150 microgrant is sufficient to reduce this aspect of material hardship [83].

The research team also considered clinical relevance, ethics, and sustainability. In many US settings, US $150 in a given month may financially support travel expenses (eg, bus, subway, rideshare, and fuel costs) to access employment, mental health, and other social services. The US $150 amount may financially support gender-affirming purchases, such as hormones, that can reduce psychological distress [84]. From an ethical standpoint, US $150 was chosen to be substantive enough for economic impact without being so high as to be coercive. Finally, community partners determined US $150 to be sustainable and scalable by community-based organizations in the United States, as noted by BTAC and demonstrated by the longevity of the Trans Lifeline microgrants program, which has distributed >US $1 million since 2018 [85].

#### Experimental Intervention: Peer Mentoring for Arm C Only

In addition to the interventions mentioned earlier, participants randomized to arm C are assigned a peer mentor who matches their gender identity (transgender man, transgender woman, or gender nonbinary). Gender matching has been found to be important to rapport building in mentoring relationships [86,87]. Peer mentoring interventions are forms of peer support that have been shown to improve mental health [88-90]. Published peer mentoring interventions vary widely in the content, structure, duration, and frequency of interactions [35,91,92]. A variety of approaches to mentoring can be effective [93-95], and telephone-delivered peer support is feasible and acceptable [96]. However, while multiple studies have tested transgender peer navigation strategies for engagement in health care [97-99], the study team found no published individual (vs group) peer mentoring interventions for mental health for TNB adults. The Healthy Divas curriculum (described in the subsequent paragraph) was selected for the CARES study because it was designed specifically for transgender people. It has been successfully implemented and found to be feasible, acceptable, and readily adaptable to diverse settings [100].

The CARES peer mentoring intervention was adapted from the Healthy Divas curriculum [101], designed for transgender women, and uses key elements of BTAC’s Akanni peer support program [102]. Healthy Divas is a manualized evidence-informed peer mentoring intervention developed by study consultant Dr Jae Sevelius and implemented at HIV care sites across the United States [100]. Based on the gender affirmation [103,104] and health empowerment [105] frameworks, the intervention improved transgender women’s HIV medication adherence through 6 sessions of strength-based peer mentoring. Guided by the 8 step method involving (1) assessment of priorities, (2) decisions on adapting, (3) administration of intervention, (4) production of adapted version, (5) topical experts, (6) integration of feedback from topical experts, (7) training staff to implement, and (8) testing the adapted intervention, ie, ADAPT-ITT [106], the CARES team, BTAC leadership, and Dr Sevelius completed a full-day workshop to adapt Healthy Divas for use with TNB people of any gender and HIV status and to focus on participant-selected goal setting. The adapted intervention was manualized by the CARES project coordinator and reviewed by the adaptation workshop participants, as well as TNB community leaders. Revisions were made based on this feedback before pilot-testing with the existing CAB, which has provided our research team with input on TNB-focused studies for several years. After pilot-testing with the CAB, the manual was finalized and used to develop training materials for peer mentors. The final peer mentoring intervention guides the participant in setting achievable goals and building their capacity to reach the goals they have selected. Each peer mentoring session addresses a specific topic (Textbox 2).

BTAC recruited, interviewed, and hired an experienced full-time peer mentor supervisor as well as 3 part-time peer mentors. The peer mentor supervisor completed the CARES peer mentoring intervention training and participated in the training of peer mentors. In addition to training on the content of the intervention and the logistics of implementation, mentor training also included skill building, such as active listening, boundary setting, and self-care [107]. Peer mentors passed the intervention competency assessment, including mock sessions, before being matched with arm C participants. The peer mentor supervisor meets with the peer mentors on a monthly basis for supervision and will make himself available throughout the study period to support the peers. In addition, mentors have access to one-on-one mental health support sessions with a nonbinary professional counselor hired by the CARES study team, as well as vouchers for a limited number of free telehealth psychotherapy sessions. Mentors meet biweekly one-on-one with their assigned participant by telephone or secure video to complete each of the 6 manualized peer mentoring sessions. Peer mentors also meet weekly with the PEC and study coordinator to troubleshoot any logistical issues that arise.

Fidelity to the peer mentorship curriculum is assessed through routine tracking of the number, duration, and content of peer interactions by the peer supervisor. In addition, participants in arm C will complete brief fidelity surveys at 3 and 6 months. All mentors have been provided with a national resource list to link mentees to supportive services as needed. Mentors track all referrals made and assess whether the participant used the referrals provided.

**Content of each Creating Access Resources and Economic Support (CARES) study peer mentoring session.**

**Session 1**

- Introductions and expectation setting
- Identify vision for future
- Introduction to goal setting
- Amplify gender affirming experience

**Session 2**

- Review vision for future
- Set attainable goals
- Identify personal strengths
- Amplify gender affirming experience

**Session 3**

- Review goals
- Discuss impact of stigma
- Strategize distress reduction strategies
- Amplify gender affirming experience

**Session 4**

- Review goals
- Identify impact of stigma
- Identify current self-care strategies
- Identify sources of support
- Set self-care goals
- Amplify gender affirming experience

**Session 5**

- Review goals
- Identify success and challenges
- Problem solve barriers to goals
- Identify additional resources
- Amplify gender affirming experience

**Session 6**

- Review goals
- Identify progress
- Revisit vision
- Set future goals
- Visualize achieving goals

### Data Analysis

#### Quantitative Data

##### Overview

Quantitative data analyses will be conducted using Stata (Stata Corp) and R (R Foundation). The distribution of all variables will be examined for outliers and to determine whether they meet the assumptions of the planned analyses. The pattern of missing data will be explored, including testing for differences in baseline variables between those with and those without missing data. Variables related to missingness will be included in the main analyses, which should yield valid inferences [108]. In the rare event of >10% missing data, we will use multiple imputation. In addition, we will conduct a series of sensitivity analyses to evaluate the robustness of conclusions with respect to departures from missing at random assumption by comparing the magnitude of the primary effect between analyses using complete data only to analyses using multiple imputation. ANOVA will be used to test whether randomization achieved balance in the baseline participant characteristics across the 3 arms. Variables in which the groups differ will be included as covariates in the main analyses. We will specify 2-sided tests and a .05 significance level. In subsequent subsections, we outline data analysis plans by study aim.

##### Aim 1

To test the efficacy of the interventions (hypothesis 1), generalized estimating equations will be used with time (baseline and 6 months), group (A, B, and C), and the group-by-time interaction included in the model. A Gaussian model will be used to examine the K6 scale. A significant group-by-time interaction would signify that the change over time differed between the 3 intervention arms. Significant group-by-time interactions will be graphed, and simple main effects will be estimated. In addition, we will examine the maintenance of the effects from 6 to 12 months using linear regression. This outcome will be the change in K6 from 6 to 12 months. Group assignments (A, B, and C) will be the main independent variable. The baseline value of the outcome will also be included in the model as a covariate. This will allow us to test whether the effect of each intervention is sustained over time in comparison to enhanced usual care. In exploratory analyses, we will repeat this set of analyses for the 10-item Drug Abuse Screening Test to examine whether the change over time in substance use differs between the 3 groups. Exploratory analyses to examine the heterogeneity of treatment effects will repeat the above-mentioned analyses stratified by race, gender, sex assigned at birth, stigma scores, and other relevant covariates. The effect sizes will be compared across the levels of each stratum to determine whether intervention effectiveness varies across groups. We will also explore whether efficacy varies by geographic location and compare the relative effectiveness of arms (A and B).

##### Aim 2

**Figure 4:**
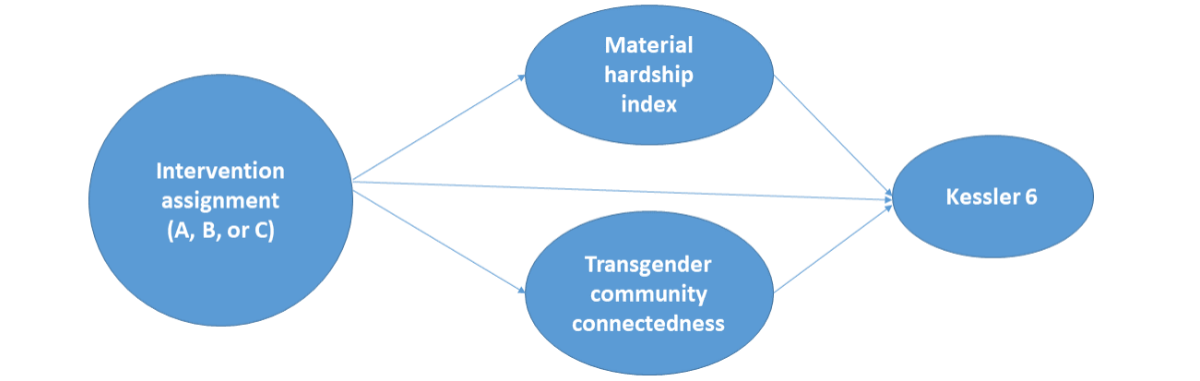
A structural equation model to be tested for aim 2.

We will examine the mechanisms by which the interventions work using structural equation modeling. Variables in the model will include a change from baseline to 6 months in the mediators (Material Hardship Index and Transgender Community Connectedness Scale) and a change from baseline to 6 months in the K6 outcome as well as group assignment (Figure 4). Structural equation modeling provides tests of the direct and indirect paths from the independent variable to the dependent variables. Of interest will be the significance tests of the indirect paths from intervention assignment through material hardship to psychological distress (hypothesis 2) and through community connectedness to psychological distress (hypothesis 2). We will examine the goodness of fit of the overall model (model chi-square, adjusted goodness of fit, comparative fit index, and standardized root mean square error of approximation).

#### Qualitative Data

##### Coding

Analysts will first compare the transcript to the digital audio recording to identify and correct any transcription errors. Then, transcripts, reflexive notes, and narrative summaries for each interview will be imported into qualitative data analysis software, such as ATLAS.ti (ATLAS.ti Scientific Software Development GmbH), to facilitate analysis. Analysis will begin with an open coding phase in which data will be read iteratively to generate analytic memos and tentative codes [109]. Open coding will be followed by systematic coding by 2 coders to ensure reliability [110].

##### Aim 3 Analysis

To take full advantage of the longitudinal nature of the data, we will apply multiple analytic approaches to the constant comparison technique [111]. We will review the narrative summaries for each participant over time to look for change within the individual. We will read across the transcripts for each period to look for themes unique to the midintervention and postintervention periods. Finally, we will read and compare all data across study arms. Analytical memos will be used to track the analytic process and describe themes that emerge. While it is not feasible for coders to be masked to the study arm, initial open coding will take place without attention to intervention assignment. Once coding is complete, we will use advanced visualization features of qualitative data management software to map out any differences in code density by study arm. Findings will be structured to best summarize participants’ experiences of material hardship and mental health as well as their perception of their assigned intervention and its efficacy. We will assess variation by race, gender, and geography.

#### Data Integration

Embedding is a type of integration that occurs when different types of data and analyses are linked at multiple points in the study and is particularly useful in intervention trials [112]. In the CARES study, qualitative data will be linked at the randomization phase when participants will be sampled from within each arm after randomization. During the intervention (month 3), qualitative data will facilitate the identification of contextual factors that could influence the trial results while also providing detailed information about the nature of the participants’ experience by the study arm. Qualitative data collected after the intervention (month 9) will facilitate the identification of changes that might be necessary for the widespread implementation of the intervention. In short, integration will help explain outcomes, improve future iterations of the intervention, and understand mediators and moderators [113]. CARES qualitative and quantitative data will also be integrated at the end of the study using result-based convergent synthesis [114]. This integration method involves analyzing qualitative and quantitative data separately and then merging results during a final synthesis [115]. Results will be merged using joint display [116], in which the interview themes will provide context for the survey results. The qualitative code density (frequency of each code) will be displayed by the study arm. Visualization will facilitate the identification of similarities and differences in participant experiences across study arms. Process data will be integrated into the joint display to visually assess any relationships among microgrant expenditures (eg, food and emergency savings fund), dose and content of the intervention (eg, peer contacts and referrals made and utilized), experience of the intervention (eg, perceived efficacy), and effect of the intervention on outcomes of interest.

## Results

Participant recruitment for the CARES study began as a “soft launch” in November 2023, when we asked our community partners to invite a few participants to screen for the study. In total, 3 participants were enrolled during the soft launch phase to ensure that study processes worked as intended before opening for full recruitment. Lessons from the implementation of this soft launch led to streamlining the enrollment process by implementing a participant self-booking option for scheduling screening and consent appointments. Full recruitment began on January 8, 2024, and, as of July 26, 2025, 138 participants have enrolled. Recruitment is expected to be completed no later than March 31, 2025, and the final study visit will take place in March 2026.

## Discussion

### Principal Findings

We hypothesize that the CARES study will demonstrate that monthly microgrants, with or without peer mentoring, will reduce psychological distress among TNB adults. Further, we anticipate that reductions in material hardship and an increased sense of connection with the transgender community will mediate these effects. We expect qualitative results to support the perceived feasibility and benefits of both microgrants and peer mentoring. This study will fill an important gap in the literature because studies on short-term microgrants in the United States are rare [117-119]. We found neither prior studies that combine microgrants with peer support nor those that aim to use financial interventions to improve the psychological well-being of TNB adults [120].

### Strengths and Limitations

The CARES study has multiple strengths. Study findings will provide important data on the efficacy of 2 community-derived, structural, and psychosocial interventions to improve mental health among TNB people in the United States and potentially mitigate some of the psychosocial harms of the COVID-19 pandemic. The community-academic partnership between BTAC, a national transgender-led organization, and a leading research university throughout the research process will ensure that rigorous data will flow directly to the community in real time. Study findings will provide useful data for BTAC and other community organizations to guide investments of their limited resources toward evidence-based programs. Findings will also have relevance for policy makers and researchers who seek to address economic vulnerability and mental health among TNB adults.

The CARES study responds to the calls for researchers to address multilevel drivers of health disparities [121] by implementing interventions that operate at the structural (economic) and interpersonal (peer mentoring) levels. In addition, this study advances the science behind minority stress theories by testing whether stigma-driven health inequities can be mitigated without intervening specifically on identity-based stigma.

However, the study has several limitations. While the team has multiple measures in place to prevent fraudulent enrollment, it is possible that people who are not TNB will misrepresent themselves to enroll in the study. To reduce this possibility, we are carefully recruiting through transgender-specific organizations, rather than through a broader approach. There is also the potential for differential attrition in the enhanced usual care arm that may lead to attrition bias. Our staff will conduct monthly check-ins for participants in this arm to enhance retention, and we are closely monitoring attrition by the study arm.

### Future Directions

The research team plans wide dissemination of study findings across policy (eg, white papers), academic (eg, scientific conferences), and community (eg, town halls) spaces to ensure results can inform future health equity approaches. Ideally, future research will build on this study’s findings to develop, refine, and test community-derived interventions to enhance TNB well-being.

## Appendix

Multimedia Appendix 1 Peer-review report by the Health Promotion in Communities (HPC) Study Section - Healthcare Delivery and Methodologies Integrated Review Group - Center for Scientific Review (National Institutes of Health, USA).

## Abbreviations

BTAC: National Black Trans Advocacy Coalition
CAB: community advisory board
CARES: Creating Access to Resources and Economic Support
GMSR: Gender Minority Stress and Resilience
IDI: in-depth interview
IRB: institutional review board
K6: 6-item Kessler Psychological Distress Scale
LITE: Leading Innovation in Trans Equity
PEC: participant engagement coordinator
TNB: transgender and nonbinary

## Data Availability

The data sets generated during this study will be available from the corresponding author upon reasonable request 1 year after the completion of data collection.
