# Supplementary material for: A Microfinance Intervention With or Without Peer Support to Improve Mental Health Among Transgender and Nonbinary Adults (the Creating Access to Resources and Economic Support Study): Protocol for a Randomized Controlled Trial
Source: JMIR Res Protoc. 2024 Aug 26;13:e63656. doi: 10.2196/63656 (PMC11384176; doi:10.2196/63656)
Supplement: Multimedia Appendix 1 [file resprot_v13i1e63656_app1.pdf]

1R01MD016755-01A1 POTEAT, TONIA

RESUME AND SUMMARY OF DISCUSSION: This resubmitted application proposes using a mixed-methods embedded 3 arm randomized controlled trial to test the efficacy of a microgrant intervention with and without peer mentoring to reduce psychological distress and promote COVID-19 risk reduction behaviors among gender minorities. The panel found the application highly significant in its focus on transgender people, a population particularly vulnerable to pandemic-related economic and mental health harms/strain. If successful, the proposed intervention's combined focus on financial and mental health could have broad applicability. The investigative team is excellent and has necessary expertise in transgender health, communications, and mixed methods to successfully carry out the project. The research environment is exceptional and bolstered by established partnerships with stakeholders, which bodes well for recruitment and retention. Reviewers found the application very responsive to prior reviews and commended the team for providing detailed justifications for changes made in this resubmission. They noted several strengths with the approach, including a robust design that is informed by substantial preliminary work, appropriate use of mixed methods, and strong analysis plan. Reviewers mentioned some weaknesses with the approach, including lack of accounting of the disparate burden of COVID across the country. There are lingering questions about why 3 arms are needed and whether the study is sufficiently powered to see differences between arms. Following discussion, the panel agreed that the strengths of the application outweighed these relatively addressable weaknesses in the approach. Overall, findings are expected to have a high impact on informing sustainable community-based responses to address COVID-19 pandemic related financial and mental health harms among gender minorities.

DESCRIPTION (provided by applicant): Transgender people experience economic and psychosocial inequities that make them particularly vulnerable to COVID-19 pandemic-related financial and mental health harms. Sustainable, multilevel interventions are needed to address these harms. Transgender-led organizations have been galvanized to provide emergency financial and peer support for transgender people negatively impacted by COVID- 19. However, the efficacy of these interventions has not been evaluated. Leveraging existing community partnerships and ongoing cohorts, the study seeks to assess the efficacy of feasible, acceptable, community-derived interventions to reduce economic and psychological harms experienced by transgender people in the wake of COVID-19. The specific aims of the project are to (1) compare the efficacy of microgrants with or without peer mentoring to reduce psychological distress; (2) examine mechanisms by which microgrants with or without peer mentoring may impact psychological distress; and (3) explore transgender participants' intervention experiences and perceived efficacy. These aims will be met by enrolling 360 transgender adults into an embedded, mixed methods, 3-arm, 12- month randomized controlled trial. Participants will be randomized 1:1:1 to the following arms: (a) a single microgrant plus monthly financial literacy education (usual care); (b) usual care plus monthly microgrants; or (c) usual care plus monthly microgrants combined with peer mentoring. All intervention arms will last for 6 months, and participants will complete semi-annual web- based surveys at 0, 6, and 12 months as well as text-based process measures at 3 and 6 months to meet Aims 1 and 2. A subset of 36 participants, 12 per arm, will complete longitudinal in depth interviews at 3 and 9 months to meet Aim 3. In addition to addressing the pressing impacts of the COVID-19 pandemic on a vulnerable health disparities population, this study will advance the science of minority stress and mental health inequities by testing interventions that operate on general stressors – i.e., material hardship and community connection – rather than minority stressors such as enacted stigma. This national, online study will address multilevel – structural and community – factors driving COVID-19 pandemic harms. Its equitable community partnership will ensure that study findings are actionable and disseminated rapidly to inform sustainable community-based responses to the COVID-19 pandemic as well as future emergencies.

CRITIQUE 1

Significance: 1
Investigator(s): 2
Innovation: 1
Approach: 3
Environment: 1

Overall Impact: Transgender people experience economic and psychosocial inequities that make them vulnerable to COVID-19 pandemic-related financial and mental health harms. This resubmission proposes to leverage existing community partnerships and ongoing national cohorts to assess the efficacy of feasible, acceptable, community-derived interventions in the form of financial support, financial counseling and peer mentoring to reduce the burden of COVID-19 experienced by transgender people. The strengths of this proposal are: it will address multilevel structural and community factors, not just short-term financial assistance; strong existing partnerships which will facilitate recruitment and retention; and excellent pilot data to support important aspects of intervention design and approach to the research. There are several moderate weaknesses, and although many appear to be addressable, they diminish the enthusiasm for the current proposal. This includes lack of detail surrounding several important components including the delivery of the micro-grants (which is especially important given the investigator with the most experience in this area is not at the prime institution), the role of differing COVID-19 burden and mandates across proposed project implementation areas/state, lack of integration and explanation about anticipated effects of intervention on COVID-19 risk behaviors, and an overly simplified explanation of the analysis plan given the complexity and inter-relatedness of many of the exploratory factors being measured.

1. Significance:

Strengths

- The current team has gathered baseline data in an observational cohort of > 1,200 transgender women which demonstrated extremely high pre-pandemic levels of important stressors as indicated by the NIMHD Minority Health and Disparities Research Framework (poverty, food insecurity, survival sex work high levels of psychological distress, traumatic stress symptom, and suicidal ideation).
- The proposed multi-level solutions of micro-grants combined with financial counseling and peer mentoring represent potential solutions not just to COVID-19 but future challenges which may also cause disproportionate hardships to transgender individuals.
- The model is based on the above framework which posits that general stress combines with minority stress leading to inequities in health and well-being. This project modifies this model to propose a *Gender Minority Stress and Resilience Model* (GMSR) which uses social connectedness to ameliorate the burden of minority stress.

Weaknesses

- There is a lack of integration of empirical or theoretical underpinnings that link COVID-19 risk behaviors to other behaviors.

2. Investigator(s):

Strengths

- PI is a certified gender health specialist, with lengthy years of experience and nearly 100 peer reviewed publications of relevance, and ongoing federally funded research. Relevant other research experience includes mixed methods and communications experience. PI has well-established relationship with Black Trans Coalition who will be an important partner in the proposed project.
- Team expertise includes psychology, social anthropology, epidemiology, communications, statistics, transgender health, reproductive health.
- Multiple investigators have experience in peer mentoring programs including with transgender groups.
- Collectively the team has specialized and highly relevant skills and expertise and are uniquely qualified to conduct the proposed research.

Weaknesses

- The only investigator with the deep experience of relevance to micro-grants does not have significant time or role on the project given the importance to the proposal.

3. Innovation:

Strengths

- Exploration of the NIMD framework for use with transgender populations and to explore stressors related to COVID-19 is innovative.
- It appears this will be among the first studies conducted in the US to generate robust data from financial assistance programs and linking this to psychosocial and related factors.

Weaknesses

- None noted by reviewer.

4. Approach

Strengths

- The project will utilize a robust design and good sample size, as well as mixed methods. They will enroll 360 transgender adults into an embedded, mixed methods, 3-arm, 12-month randomized controlled trial which will allow the assessment of the assess the efficacy of microgrants with or without peer monitoring and understand the impact on distress and understand the experience and perceived utility among the participants. Participants will be randomized 1:1:1 to the following arms: (a) a single microgrant plus monthly financial literacy education (usual care); (b) usual care plus monthly microgrants; or (c) usual care plus monthly microgrants combined with peer mentoring to distinguish between the financial and peer component impact.
- Coalition already delivering microgrants so the feasibility of the recruitment and retention is high.
- Previous significant experience with collecting longitudinal data in the proposed study population.

- The integration of financial counseling is based on important findings from their preliminary work and therefore driven by community need.
- Data collection and instruments are well-planned and thought out with sound rationale.
- Excellent study power calculations, based on 20% effect in hardship (primary outcome of study).
- Though the study does not have a true control, it incorporates a group X time interaction to improve robustness of comparison.
- Integration of proposed qualitative and quantitative components well-described.
- Calculation of amount of microgrant is well-justified.
- Fidelity for peer support component appears high.

Weaknesses

- (Minor) The analysis plan does not account for differing COVID burdens and mandates across data collection sites, which could contribute to pandemic-related distress.
- (Moderate) There are a very high number of exploratory factors and the analysis plan does not address how to approach this with enough detail (e.g., many are inter-related, such as anxiety with multiple others). Also, many demographic and related characteristics are not explicit in the analysis plan.

5. Environment:

Strengths

- Academic Center has multiple relevant resources and numerous project resources.
- Strong partnership with the National Black Trans Advocacy Coalition, who has a national office in Texas

Weaknesses

- Director of Transgender Research is not described in Facilities and Resources Section, which seems significant resource for the project.

Study Timeline:

Strengths

- None noted by reviewer.

Weaknesses

- None noted by reviewer.

Protections for Human Subjects:

Acceptable Risks and/or Adequate Protections

- Organization already providing micro-grants based on need so individuals will not be excluded from receiving the funds if not part of the study

Data and Safety Monitoring Plan (Applicable for Clinical Trials Only):

Acceptable

Inclusion Plans:

- Sex/Gender: Distribution justified scientifically
- Race/Ethnicity: Distribution justified scientifically
- Inclusion/Exclusion Based on Age: Distribution justified scientifically
- Population includes transgender men, transgender women, and gender nonbinary

Vertebrate Animals:
Not Applicable (No Vertebrate Animals)

Biohazards:
Not Applicable (No Biohazards)

Resubmission:

- The investigators replied thoroughly to the comments from previous reviewers and provided justification if changes were not made. However, some of the issues with the psychosocial factors and analysis plan were still noted as lacking some important components as noted in this review.

Resource Sharing Plans:
Acceptable

Budget and Period of Support:
Recommend as Requested

CRITIQUE 2

Significance: 2
Investigator(s): 1
Innovation: 3
Approach: 3
Environment: 1

Overall Impact: This strong investigative team aims to (1) compare microgrants with or without peer mentoring to reduce psychological distress (2) examine mechanisms by which microgrants or unconditional cash transfers with or without peer mentoring may impact psychological distress; and (3) explore transgender participants' intervention experiences and perceived efficacy. In this RCT, 360 transgender adults will be assigned to a single microgrant plus monthly financial literacy education (usual care); usual care plus monthly microgrants; or usual care plus monthly microgrants combined with peer mentoring. the investigators were generally responsive to the previous reviewers' concerns. Strengths include the study's significance; there's a lack of

intervention outcome studies in transgender people although they've demonstrated susceptibility to mental health disorders and poverty, and the investigators propose an RCT within the context of existing programming. Other strengths include state of the art resources, a strong environment, established relationships of investigative team with stakeholders, they've conducted several pilot studies demonstrating the likelihood they can complete the study, and steps to assist in quality control. A minor weakness is that in response to the reviewers' original concerns, the investigators indicate the sample size required to identify a significant effect when comparing intervention arms to each other is quite large and not feasible within the R01 budget. Effect sizes for the three groups will be examined, but significant differences between the two intervention arms are not hypothesized, thus it is unclear why three arms are necessary.

1. Significance:

Strengths

- There is strong support for the importance of intervention development in transgender individuals.

Weaknesses

- None noted by reviewer.

2. Investigator(s):

Strengths

- PI and investigative team have extensive work together with transgender individuals, project roles of investigative team are complementary.

Weaknesses

- None noted by reviewer.

3. Innovation:

Strengths

- Examining how financial assistance impacts mental health of transgender individuals is innovative.

Weaknesses

- None noted by reviewer.

4. Approach:

Strengths

- Recruitment is comprehensive and well-planned
- quantitative and qualitative data
- experimental interventions are innovative
- quality control
- methods of managing missing data.
- relevant pilot studies

Weaknesses

- The research team is large, although they appear to have a history working together efficiently.
- Significant differences between the two experimental arms are not hypothesized to occur.

5. Environment:
Strengths
- The partnerships are well-specified, and resources are outstanding.

Weaknesses
- None noted by reviewer.

Study Timeline:
Strengths
- The timeline is appropriate

Weaknesses
- None noted by reviewer.

Protections for Human Subjects:
Acceptable Risks and/or Adequate Protections
- Very comprehensive

Data and Safety Monitoring Plan (Applicable for Clinical Trials Only):
Acceptable
- appropriate

Inclusion Plans
- Sex/Gender: Distribution justified scientifically
- Race/Ethnicity: Distribution justified scientifically
- Inclusion/Exclusion Based on Age: Distribution justified scientifically

Vertebrate Animals:
Not Applicable (No Vertebrate Animals)

Biohazards:
Not Applicable (No Biohazards)

Resource Sharing Plans:
Acceptable

Budget and Period of Support:
Recommend as Requested

CRITIQUE 3

Significance: 1
Investigator(s): 1
Innovation: 2
Approach: 2
Environment: 1

Overall Impact: This proposal addresses a highly significant issue of mental and economic consequences of the covid-19 pandemic among transgender people. The application will use a mixed-methods embedded 3 arm RCT to examine the utility of microgrants and peer mentoring to improve mental health outcomes, including mechanisms to achieve outcomes and intervention experience. Overall, the scientific rigor is strong, this is a strong team, and the approach is justified to carry out the aims. The proposers were highly responsive to prior critiques. Overall, this proposal will have high impact.

1. Significance:

Strengths

- Transgender people face significant structural barriers to health and social prosperity, including economic opportunity.
- Focus on microgrants supported by strong scientific premise.
- Rigor of prior research and preliminary data by the study team is strong.

Weaknesses

- None noted by reviewer.

2. Investigator(s):

Strengths

- This is a strong team with complimentary expertise and track record to carry out the work.

Weaknesses

- None noted by reviewer.

3. Innovation:

Strengths

- The study will advance the field by incorporating peer support in an economic intervention and testing psychological outcomes. This is novel and has not been rigorously examined.

Weaknesses

- None noted by reviewer.

4. Approach:

Strengths

- Strong theoretical framework and preliminary data to support the proposal.
- The study design is appropriate.
- Community support and partnerships are strong.
- Overall adequate measures and sampling approach.

Weaknesses

- None noted by reviewer.

5. Environment:

Strengths

- Strong research environment to support the study aims.

Weaknesses

- None noted by reviewer.

Study Timeline:

Strengths

- Adequate.

Weaknesses

- None noted by reviewer.

Protections for Human Subjects:

Acceptable Risks and/or Adequate Protections

- adequate

Data and Safety Monitoring Plan (Applicable for Clinical Trials Only):

Acceptable

- adequate

Inclusion Plans

- Sex/Gender: Distribution justified scientifically
- Race/Ethnicity: Distribution justified scientifically
- Inclusion/Exclusion Based on Age: Distribution justified scientifically
- adequate

Vertebrate Animals:

Not Applicable (No Vertebrate Animals)

Biohazards:

Not Applicable (No Biohazards)

Resubmission:

- The proposal was highly responsive to prior critiques.

Resource Sharing Plans:

Acceptable

Budget and Period of Support:

Recommend as Requested

THE FOLLOWING SECTIONS WERE PREPARED BY THE SCIENTIFIC REVIEW OFFICER TO SUMMARIZE THE OUTCOME OF DISCUSSIONS OF THE REVIEW COMMITTEE, OR REVIEWERS' WRITTEN CRITIQUES, ON THE FOLLOWING ISSUES:

PROTECTION OF HUMAN SUBJECTS: ACCEPTABLE

INCLUSION OF WOMEN PLAN: ACCEPTABLE

INCLUSION OF MINORITIES PLAN: ACCEPTABLE

INCLUSION ACROSS THE LIFESPAN: ACCEPTABLE
